# Transfer‐Printed Wrinkled PVDF‐Based Tactile Sensor‐Nanogenerator Bundle for Hybrid Piezoelectric‐Triboelectric Potential Generation

**DOI:** 10.1002/smll.202502767

**Published:** 2025-05-08

**Authors:** Kamal Kumar Meena, Injamamul Arief, Anik Kumar Ghosh, André Knapp, Mirko Nitschke, Andreas Fery, Amit Das

**Affiliations:** ^1^ Leibniz‐Institut für Polymerforschung Dresden e.V. Hohe Straße 6 D‐01069 Dresden Germany; ^2^ Center for Advancing Electronics Dresden (cfaed) Technische Universität Dresden Helmholtzstraße 18 D‐01069 Dresden Germany; ^3^ Tampere University Tampere 33720 Finland

**Keywords:** imprinted PVDF, piezoelectric‐triboelectric hybrid potential, self‐powered tactile sensor, transfer printing, triboelectric nanogenerator

## Abstract

Triboelectric sensors are known for their ultrahigh sensitivity and wide‐range detectability of tactile force/pressure, all while being self‐powered. However, the energy harvesting efficiency of triboelectric nanogenerators (TENGs) is often limited by relatively low output power density, when compared to other state‐of‐the‐art microgenerators. To address this challenge and achieve high force/pressure detection while maintaining excellent tactile resolution, a hybrid nanogenerator is proposed that comprises of both triboelectric and piezoelectric components within a ferroelectric polyvinylidene fluoride (PVDF) polymer matrix. To enhance tactile sensitivity, a coupled transfer printed‐spin coating technique is introduced to imprint wrinkled silicone structuring with tunable periodicity and amplitude directly onto PVDF. The hybrid output voltage of the wrinkled PVDF‐based TENG utilizing the ferroelectric β phase of PVDF (FE‐TENG_5) shows an impressive ≈200% increase compared to pristine FE‐TENG. The highest power density (0.9 mW cm^−2^) corresponds to FE‐TENG with the periodicity of 5 µm. Remarkably, the imprinted FE‐TENGs can detect even the slightest tactile force (<2 N), while the hybrid mechanism ensures a broad force sensing range, extending up to 100 N before saturation. This exceptional performance establishes the imprinted PVDF‐based FE‐TENG as a versatile tactile sensing platform for a range of cutting‐edge applications, particularly in electronic skin and next‐generation microelectronics.

## Graphical abstract

We introduce a novel lithography‐free method that combines PDMS‐based surface wrinkling with subsequent PVDF imprinting to produce ultrasensitive patterned surfaces. The controlled wrinkle periodicity enhances triboelectric nanogenerator efficiency and tactile sensitivity, achieving scalability and cost‐effectiveness of the fabrication. This approach integrates high‐resolution microstructures, yielding potential for energy harvesting and advanced sensing applications in wearable technologies.

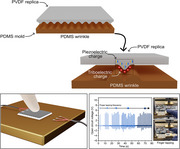

## 1 Introduction

The human sense of touch is incredibly detailed and important for interacting with the world and detecting small changes in pressure and texture. Emulating this level of sensitivity in electronic devices has become decisive for emerging fields such as wearable electronics, advanced robotics, and human–machine interfaces.^[^ ^1^, ^2^, ^3^ ^]^ However, powering these devices efficiently and sustainably remains a challenge.^[^ ^4^ ^]^ In this context, triboelectric nanogenerators (TENGs),^[^ ^5^ ^]^ capable of converting ambient mechanical energy directly into electrical signals, present an attractive self‐powered solution. Whereas, the term “nanogenerator” is commonly used to refer nanoscale interfacial charge generation rather than literal device dimensions.^[^ ^6^, ^7^ ^]^ Over the years TENGs have positioned itself as promising candidates for powering small electronics and sensors.^[^ ^6^ ^]^

These devices can be fabricated using any of a range of possible materials, including inorganic, organic (e.g., polymers), and hybrid systems.^[^ ^8^, ^9^, ^10^ ^]^ High piezoelectric coefficients are sought after when making energy conversion devices, and inorganic materials possess many of these qualities, such as barium titanate (BaTiO_3_) and zinc oxide (ZnO), which can convert energy with up to 99% efficiency.^[^ ^11^, ^12^, ^13^ ^]^ However, the inherent brittleness and rigidity of these materials restrict their use due to a lack of flexibility, mechanical adaptability, and biocompatibility, all of which are key interests for wearable technology and tactile sensing.^[^ ^14^, ^15^ ^]^ Conversely, organic materials such as thermoplastic polymers exhibit obvious advantages.^[^ ^16^ ^]^ In particular, polyvinylidene fluoride (PVDF) is often considered to be a highly sought after nanogenerator and transducer material due to the superior mechanical advantages that couldn't be found in inorganic crystals and amorphous materials.^[^ ^9^ ^]^

Among various materials explored for TENGs, PVDF and its copolymers have instigated particular attention because of their unique combination of properties.^[^ ^17^ ^]^ PVDF is highly electronegative and can exhibit strong piezoelectric and pyroelectric responses when crystallized in its polar phases (predominantly β). The superior performance of its ferroelectric β‐phase can further be augmented through mechanical stretching, and electrical poling,^[^ ^18^ ^]^ which align molecular dipoles and induce robust remnant polarization. Optimizing α/β/γ phase composition via thermal processing has also been shown to drastically enhance energy storage and discharge efficiency in pure PVDF systems.^[^ ^19^ ^]^

The performance of PVDF‐based nanogenerators is primarily (in addition to triboelectric effect) associated to the degree of dipole alignment and the material's ferroelectric polarization.^[^ ^20^ ^]^ As explained, β‐phase, in particular, exhibits a strong net dipole moment resulting in a pronounced piezoelectric response.^[^ ^21^, ^22^ ^]^ When engineered at micro‐ or nanoscale, the surface geometry of PVDF films introduces significant local curvature, which amplifies the effective electric field due to the so‐called “lightning rod effect,” a phenomenon also witnessed in hybrid tactile sensors to boost sensitivity and energy output through hierarchical structuring.^[^ ^23^, ^24^, ^25^ ^]^ This localized field enhancement not only promotes surface charge accumulation under triboelectric contact but also synergizes with the ferroelectric dipole orientation, eventually boosting hybrid charge generation.^[^ ^26^, ^27^ ^]^ By carefully engineering these microstructural features, it becomes possible to manipulate interfacial charge density and energy conversion efficiency without external poling—a crucial step toward developing high‐performance, scalable hybrid TENGs.^[^ ^28^ ^]^

A critical factor influencing TENG performance is the surface morphology of the triboelectric layer.^[^ ^29^, ^30^ ^]^ For PVDF‐based nanogenerators, controlling surface texture at micro‐ to nanoscales is essential to optimize dipolar orientation, surface charge density, and energy conversion efficiency.^[^ ^31^, ^32^, ^33^ ^]^ Accordingly, various surface structuring techniques have been investigated for PVDF, including electrospinning to create nanofibrous mats, laser ablation to directly pattern micro‐features, thermal embossing or nanoimprinting, and chemical etching or nanoparticle embedding to increase roughness.^[^ ^34^, ^35^, ^36^, ^37^, ^38^ ^]^ Each of these methods, however, suffer from drawbacks, such as, complex fabrication processes, limited scalability, or material degradation, particularly in techniques relying on lithographic or mold‐based patterning.^[^ ^7^, ^39^, ^40^ ^]^ For instance, while laser ablation possesses excellent patterning capabilities, it risks thermally damaging the polymer surface.^[^ ^41^ ^]^ Similarly, chemical etching is a cost‐effective solution for surface modification but raises environmental and safety concerns due to aggressive chemicals.^[^ ^42^, ^43^, ^44^ ^]^

In recent years, template‐assisted microstructuring has proven effective for enhancing TENG outputs without extensive lithographic measures.^[^ ^45^ ^]^ By introducing deliberate surface patterns on one or both contacting surfaces, the effective contact area and local field intensity can be enhanced.^[^ ^6^, ^46^ ^]^ For example, Tcho et al. examined the effect of well‐ordered microstructures on a PDMS triboelectric layer, comparing dome‐shaped versus pillar‐shaped surface patterns.^[^ ^6^ ^]^ The dome‐patterned TENG showed higher force sensitivity than the pillar‐patterned one, whereas the latter exhibited better durability under repetitive stress.^[^ ^7^, ^25^ ^]^ These results highlight how geometric shape and scale of surface features critically influence triboelectric performance and mechanical robustness.^[^ ^6^ ^]^ For instance, Fatma et al. fabricated mechanically robust PVDF films by solution casting under controlled humidity: a film cast at low humidity (10% RH) had a rougher surface, higher β/γ‐phase content, and yielded *V*_OC_ ≈ 410 V, *I*_SC_ ≈ 14.8 µA, and power output ≈3.5 mW (0.136 mW cm⁻^2^)—about threefold higher power density than a film cast at 54% RH.^[^ ^47^ ^]^ They demonstrated this high‐output PVDF TENG powering >100 LEDs via foot stomping and functioning as a self‐powered wireless motion sensor, functioning similarly to hydrogel‐based^[^ ^48^ ^]^ supercapacitor systems. In another approach, Shafeek et al. doped PVDF with silicon carbide (SiC) nanoparticles to enhance its ferroelectric phase fraction and dielectric constant, resulting in a composite film TENG that achieved open‐circuit voltages up to ≈850–1100 V and *I*_SC_ ≈20 µA (at a few Hz operation).^[^ ^44^ ^]^ Mao et al. further addressed durability challenges by designing a CB/Fe₃O₄/PDMS‐based rotary TENG with rolling microarray contact, maintaining stable output after 100 000 cycles while achieving power densities up to 16.2 mW m^−^^2^.^[^ ^49^ ^]^ These diverse studies clearly show that surface‐structured or composite‐enhanced PVDF‐based TENGs can outperform flat PVDF films or other smooth polymer TENGs.

Despite progress in the above approaches, challenges remain in achieving high sensitivity at low pressures and maintaining output over a broad pressure range, all via a scalable low‐cost fabrication route. In addition, many prior works focus on either triboelectric or piezoelectric enhancements alone, without completely using both. Here, we address these gaps by introducing a lithography‐free microstructuring technique based on wrinkling to imprint PVDF with tunable micro‐crests and troughs.^[^ ^50^ ^]^ Unlike conventional top‐down lithographic routes, that can be costly, environmentally taxing due to high energy input and challenging to scale, wrinkling offers a self‐organized approach to pattern large areas (from cm^2^ to m^2^) at low cost and with minimal chemical waste.^[^ ^51^ ^]^ Specifically, we use H_2_ plasma or UV/ozone surface treatments to stiffen the top surface of silicone substrate, then induce mechanical strain to from wrinkles.^[^ ^52^, ^53^, ^54^ ^]^ The wavelength, amplitude, and morphology of these wrinkles can be “programmed” by controlling the applied strain, layer thicknesses and elasticity contrasts.^[^ ^52^, ^55^ ^]^ We then spin‐coat PVDF onto these reusable wrinkled silicone template, effectively transfer‐printing an inverse replica microstructure onto PVDF. In this way we create a ferroelectric TENG (FE‐TENG) that benefits from both the inherent piezoelectricity and an enhanced triboelectric effect from its microstructured topography.^[^ ^56^ ^]^

This work differentiates itself from prior studies in both the structuring technique and the device functionality. Unlike classical top‐down structuring or random roughening, we produce highly regular microstructures at minimal cost.^[^ ^50^, ^57^ ^]^ Moreover, the wrinkle morphology can be fine‐tuned to achieve unprecedented improvements in triboelectric output, (including a force sensitivity down to 2 N, making it among the most sensitive tactile sensors reported to date) while maintaining a broad operational range up to 100 N. In addition, our results show that the hybrid design can effectively decouple simultaneously presented stimuli through advanced electron flow analysis and temporal response characterization, making signals clearer and more discriminable. Through these notions, the present study offers a unique contribution relative to existing literature, providing a scalable route to high‐performance PVDF based TENGs for self‐powered sensing.

## 2 Result and Discussion

### 2.1 Fabrication of Ultrasensitive Imprinted PVDF via “Surface Wrinkling”

**Figure 1:**
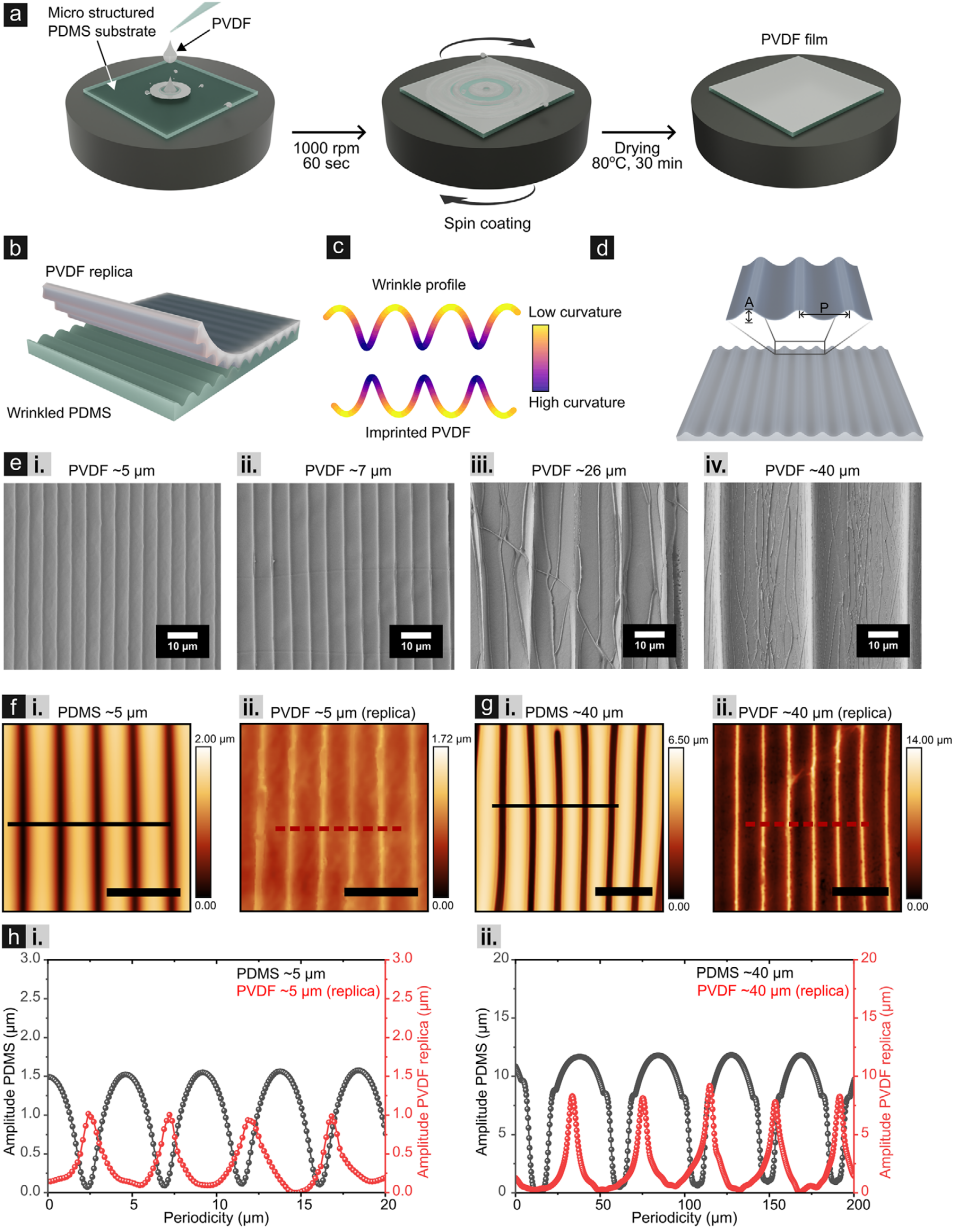
Fabrication, structural replication, and morphological characterization of wrinkled PVDF surfaces. a) A schematic of the spin coating process used for the formulation of imprinted PVDF films. b) An illustration depicting the wrinkled PDMS structure as a template and the imprinted PVDF structure as replica. c) Pictorial demonstration of curvature for wrinkled and imprinted PVDF. d) Key morphological parameters of the imprinted PVDF structure, with *P* representing the periodicity (distance between two consecutive peaks) and *A* denoting the amplitude (vertical distance between crest and valley of the profile). e) SEM images of the imprinted PVDF surfaces exhibiting approximate periodicities of ≈5 µm (i), ≈7 µm (ii), ≈26 µm (iii), and ≈40 µm (iv). f) Confocal microscopic images of the wrinkled PDMS template and the corresponding imprinted PVDF patterns, showing periodicities of 4.68 ± 0.11 µm (i) and 4.72 ± 0.66 µm (ii), as well as 39.86 ± 5.77 µm g‐i) and 40.07 ± 5.30 µm g‐ii), respectively. Scale bars: 10 µm in (f) and 100 µm in (g). h) Line profile comparisons between the wrinkled PDMS template and the imprinted PVDF structures, highlighting periodicities around ≈5 µm (i) and ≈40 µm (ii). The locations of the extracted profiles are indicated in (f,g).

As highlighted in the introduction, wrinkling based surface structuring offers significant advantages over conventional top‐down lithographic methods, including reduced fabrication complexity, cost effectiveness and scalability.^[^ ^58^ ^]^ Polydimethylsiloxane (PDMS) is selected as the template material, owing to its compatibility with controlled H_2_ plasma and UV/ozone surface treatments.^[^ ^52^, ^58^ ^]^ These treatments allow for fine‐tuning of key wrinkle parameters, including wavelength, amplitude, and shape, ensuring well‐defined and optimized wrinkled structures.^[^ ^59^ ^]^ These structured surfaces subsequently act as reusable template for spin‐coating homogeneous solution of PVDF dissolved in 99.5% N,N‐dimethylformamide (DMF). **Figure** **1**a schematically illustrates the detailed spin coating process, highlighting the formation of the imprinted PVDF. Details about the spin coating parameters can be found in the experimental section.

The choice of solvent is critical, since solvents with a higher solubility parameter tend to swell PDMS more, while DMF, having a solubility parameter of 12.1 cal^1/2^ cm^−3/2^ can induce a moderate swelling in PDMS.^[^ ^60^ ^]^ Although, DMF is permeable through the PDMS bulk, the stiffer top layer with higher crosslink density tends to have lower porosity.^[^ ^61^ ^]^ Thus reduced swelling of the top layer is expected when compared to the bulk, thus keeping the shape of the individual wrinkles as close as possible with the original one. Figure 1b depicts this demolding step, wherein the PVDF thin film (replica) is separated from the PDMS (template). It is important to mention that we predict an ideal transfer of wrinkling morphology onto the PVDF film, thus the imprinted PVDF (replica) is represented as an ideal transferred version of the wrinkled PDMS template in Figure 1c. The predicted transfer accuracy is presented in terms of curvature in the following section. Notably, wrinkled surfaces are inherently asymmetric, with “valleys” having higher curvature compared to the “peaks” (Figure 1c). The zoomed‐in view of the imprinted PVDF is characterized by periodicity (*P*), and amplitude (*A*), inducing a direct comparison with the wrinkled PDMS template (Figure 1d).

Surface curvature is a critical factor influencing the distribution of the triboelectric charges on wrinkled substrates. By enhancing the overall contact area and interaction between surfaces, curvature optimization boosts the energy harvesting efficiency of TENGs.^[^ ^62^ ^]^ This asymmetry is advantageous for triboelectric applications, as the sharper peaks in the imprinted PVDF structure concentrate stress, thereby enhancing the triboelectric charge density.^[^ ^26^, ^63^ ^]^ As the periodicity changes, so does the curvature distribution across the surface, affecting the local electric field and, consequently, the charge distribution. This variability introduces opportunities for intricate surface design that could optimize triboelectric performance. However, it should be noted that the patterning process can introduce irregularities such as y‐shaped defects and cracks, due to the strain‐release mechanism inherent in the wrinkling process (Figure S1d, Supporting Information). Thus, the same can be expected to be replicated in the imprinted PVDF. Although these can be minimized through controlled strain release patterns, to some extent it remains unavoidable.^[^ ^24^ ^]^ Expectedly, such irregularities do not significantly affect the triboelectric charge distribution, ensuring the effectiveness of the triboelectric nanogenerator.

We validated the effectiveness of the fabrication process, and the triboelectric performance of imprinted PVDF films across a broader range of periodicities. Specifically, periodicities ranging from microscale (≈5 µm) to sub‐millimeter scales (≈40 µm) are taken into consideration. It is important to note that an offset between the targeted and the produced periodicity of wrinkles in PDMS is observed, due to intrinsic material properties, processing conditions, and other physical constraints.^[^ ^50^ ^]^ While an in‐depth discussion on this offset is beyond the scope of the current article. Choice of periodicities from such a wide range allows for a systematic evaluation of the influence of curvature, electric field distribution, and the local contact area on triboelectric charge generation. Furthermore, the chosen periodicities reflect scales relevant to both high‐performance microscale devices and practical, scalable applications, enabling the study to fundamental insights and real‐world relevance. SEM micrographs of the imprinted PVDF are presented in Figure 1e The free‐standing nature of the PVDF films required careful mounting during imaging to minimize distortion (detailed in Figure S2, Supporting Information). The SEM images of imprinted PVDFs show a clear demarcation of the increased periodicity from left to right (i–iv), and the associated wrinkled PDMS templates are presented in Figure S3 (Supporting Information). The SEM images (Figure 1e) after spin coating confirm the uniformity for PVDF deposition and the integrity of the underlying wrinkled patterns. Further quantitative evaluation of the transfer process is presented as follows.

To quantitatively evaluate the accuracy of the process, surface topographic images obtained from confocal microscopy (Figure 1f,g) have been evaluated (Figure 1h–i,ii). Further on, the same has been used to calculate the periodicities and amplitude of the wrinkled PDMS template and imprinted PVDF, and has been listed in Table S1 (Supporting Information). A python script has been used to evaluate the values over three micrographs and the averaging has been done over those to have more generic understanding.^[^ ^53^ ^]^

Peak‐to‐peak comparisons (Figure 1h) of these parameters assessed the uniformity and fidelity of the transferred patterns. For simplicity, comparative study for the ≈ 5 µm and ≈40 µm periodic imprinted PVDF structures is shown in Figure 1h, while results of the remaining samples are elaborated in Figure S4 (Supporting Information). To ensure a more generic quantification, four random peaks have been chosen from the wrinkled PDMS template and the imprinted PVDF substrate. Periodicity and amplitude obtained from these peaks have been tabulated in Table S2 (Supporting Information) and the same values have been used to calculate further parameters. When considering all the imprinted PVDF substrates (Table S3, Supporting Information), except the case of ≈26 µm, amplitude ratios remain below 1 for all the samples (≈5 µm, ≈7 µm, and ≈40 µm), indicating under‐replication of the amplitude during the transfer process (Table S3, Supporting Information). This under replication can be attributed to several factors, such as insufficient penetration of the PVDF into deeper valleys, in addition the annealing step could reduce the ability to conform wrinkling structures: thus, affecting the amplitude replication.

As mentioned, samples show under‐replication, although the accuracy for the amplitude transfer ranges from 64.75% (≈5 µm) to 70.60% (≈40 µm) except for the ≈26 µm case. At the same time, the coefficient of variation (CV) for amplitude is low for smaller periodicities (≈5 µm: 3.33%, ≈8 µm: 3.51%) and increases slightly for the largest (≈40 µm: 5.41%), indicating overall consistent amplitude transfer for wrinkles (Table S4, Supporting Information). Also, the cooling process may have led to slight relaxation of the PVDF film, mitigating thermal stresses, particularly for ≈40 µm samples, thus contributing to minor variability. In terms of periodicity transfer, the periodicity ratios are close to 1 for all the samples. Accuracy values range from 87.20% (≈7 µm) to 94.79% (≈5 µm), indicating high fidelity as well as robust periodicity transfer for small and large periodic samples. At the same time the CV values remain consistent too (≈2.55%–3.36%), implying reliability of the replication process.

In case of ≈26 µm sample, the behavior is anomalous, probably due to the fabrication issues, such as bi‐sinusoidal or branched behavior.^[^ ^64^ ^]^ Its amplitude ratio of 1.74 indicates over‐replication and the amplitude accuracy of 25.68% is significantly lower than the other samples. Such deviation can probably be attributed to fabrication irregularities. Interestingly, the periodicity ratio (1.08) and accuracy (91.80%) remain within the acceptable limits, suggesting that the primary issue is with the amplitude replication. Notably, the higher periodicity accuracy, despite the amplitude issues, highlights the robustness of the fabrication process in preserving the spatial patterns. Overall, these findings demonstrate that wrinkling and spin coating process provides a robust method for preparing high‐performance TENG devices, supporting its potential for scalable production.

**Figure 2:**
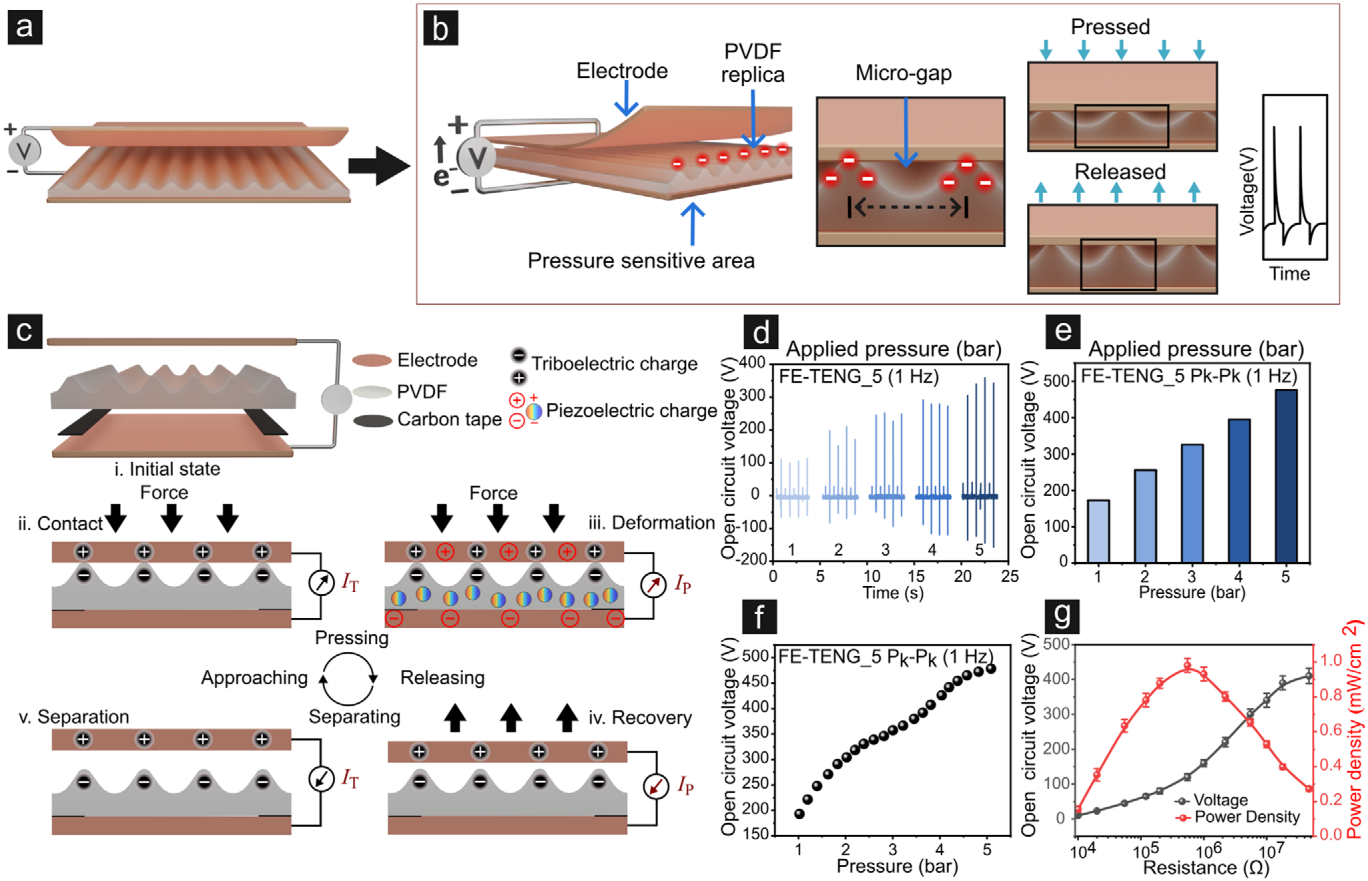
Schematic and performance analysis of the imprinted PVDF nanogenerator. a) Illustration of the multimodal imprinted PVDF sensor. b) Representative voltage‐time data demonstrating the sensor's response under cyclic pressure and the associated charge accumulation dynamics. c) Stepwise operational mechanism detailing (i) initial state, (ii) contact initiation, (iii) deformation, (iv) recovery, and (v) separation, indicating generation of both piezoelectric and triboelectric charges in response to mechanical force. d,e) Voltage responses measured under varied cyclic pressures (1–5 bar) at a constant frequency of 1 Hz. f) The peak‐to‐peak voltage demonstrates a linear increase with applied pressure, thereby improving energy harvesting capabilities under different load conditions (1–5 bar). g) Optimization analysis of FE‐TENG_5 reveals how variations in external resistance (20 kΩ–47 MΩ) affect both the voltage and power density, ultimately identifying the conditions that yield the maximum power density.

The selected geometries were designed to optimize charge distribution and density for triboelectric performance enhancement. The formation of such an imprinted PVDF sensor is depicted in **Figure** **2**a,b. It is to note that surfaces with varying local curvatures (for example, sharp peaks) amplify local electric fields, concentrate triboelectrically formed charges, and increase the efficiency of energy harvesting. Furthermore, high curvature geometries increase localized charge density in an intensified electric field, whereas broader geometries enhance uniform charge distribution, both of which make curvature an important parameter in realizing optimized performance. As depicted in Figure 2b, in the pressed state the imprinted PVDF deforms, minimizing the micro‐gaps and increasing the effective contact area. Upon release, the imprinted PVDF recovers to their original configuration, restoring the micro‐gaps and facilitating charge accumulation. This cyclical deformation and recovery process is crucial for continuous energy generation for the sensor. By combining these geometries, sensors capable of sensing multiplicity of physical inputs such as pressure, bending, and proximity are constructed. While fabrication‐induced defects or variability in local curvature present challenges, thus such hybrid designs show promise for robust, scalable, and adaptive sensor technologies.

### 2.2 Device Structure and Operational Principle

The sensor's pressure/force sensitivity stems primarily from coupled piezoelectric‐triboelectric hybrid mechano‐electric phenomenon. In this process, piezoelectric transduction occurs when mechanical stress within a material without structural symmetry induces electrical polarization. Whereas, triboelectric voltage generation result from contact electrification and electrostatic induction during repeated contact and separation cycles (Figure 2c).^[^ ^65^ ^]^ The top and bottom of the imprinted PVDF film produce surface charges with opposite polarities when the applied pressure reduces the micro‐gap present due to the wrinkled topography of imprinted PVDF film. As the pressure is released, allowing the micro‐gap to be restored, electrons move through the external circuit until the accumulated charges reach equilibrium.^[^ ^66^ ^]^ The imprinted PVDF film responds to pressure by collapsing the micro‐gaps and causing electron flow in the opposite direction to balance the charge. The electrical signal in terms of open circuit voltage is produced as a result of internally hybridized triboelectric output coupled with the piezoelectric effect of the intrinsic PVDF (flat substrate). A typical comparison of electrical output for pristine and imprinted FE‐TENGs is shown in Figure S5 (Supporting Information). This has to be noted that hybridization of piezoelectric and triboelectric mechanism in transducers is now regarded as an efficient route to improve electromechanical performance. Several hybrid nanogenerators have been reported in past years to demonstrate facile mechano‐electrical conversion.^[^ ^13^, ^66^, ^67^, ^68^ ^]^ Piezoelectric effect defines a change in dipolar polarization in ferroelectric polymers (e.g., PVDF) when subjected to external stresses.^[^ ^69^, ^70^ ^]^ Triboelectricity, on the other hand, is the coupled effect of electrostatic induction and contact electrification when two dielectric substrates with variable work functions come in contact.^[^ ^70^, ^71^ ^]^ In vertical contact‐separation device, continuous alternating hybrid voltammograms are, therefore, generated for FE‐TENGs in response to repeated contact‐separation movements.

Figure 2d shows real‐time pressure‐driven hybrid piezoelectric‐triboelectric voltammograms of FE‐TENG_5 involving imprinted PVDF (5 µm wavelength of periodicity) film under a broad range of applied vertical pressures (pneumatic motor pressure varied between 1 and 5 bar and at 1 Hz, sensor dimensions ≈1.5 cm × 1.5 cm). At lower (1 bar) and higher (5 bar) vertical contact pressures, FE‐TENG_5 exhibits variable sensitivities, producing open circuit (peak‐to‐peak) voltages of 185 and 490 V, respectively (Figure 2e,f). The vertical pressure sensitivity at a constant frequency of 1 Hz shows a nearly linear trend in terms of electrical output and has been observed in previously reported FE‐TENGs. The initial rise in voltage output is shown to be higher, followed by a narrow plateau (2.5–3.5 bar) and subsequent sharp increment till the saturation point at very high applied pressure (>5 bar). As the frequency is kept constant, the linear correlation to mechanical pressure exemplifies the mechano‐electric transduction of the FE‐TENG, which is a product of hybridized piezoelectric and triboelectric voltage outputs. Although the individual signals cannot be independently extracted in the current setup, previous literature supported a predominantly TENG‐based mechanism where, piezoelectric part contributes only slightly.^[^ ^71^ ^]^ More comprehensive discussion on respective contribution is presented in the succeeding section.

It was observed that the amplitude of the output voltage (*V*_OC_) across the external load resistance increased with increasing resistance and became saturated at a high resistance of 47 MΩ. Similarly, the electrical output power was also measured. Additionally, to quantify the output power densities of FE‐TENGs, the voltage and current were measured under varying external load resistance (20 kΩ–47 MΩ, Figures 2g and S6, Supporting Information). The maximum power density of the FE‐TENG_5 was evaluated to be 0.9 mW cm^−2^, under the matching load of 10 MΩ. This implies that the internal resistance of the FE‐TENG_5 was approx. 10 MΩ, based on matching load conditions for maximized output. We have also estimated the transferred charge per cycle by integrating the short‐circuit current over time (current waveform). The calculated value was 0.231 µC for FE‐TENG_5 and at 1 Hz.

### 2.3 Tactile Pressure Sensing Performance of Imprinted PVDF FE‐TENGs

**Figure 3:**
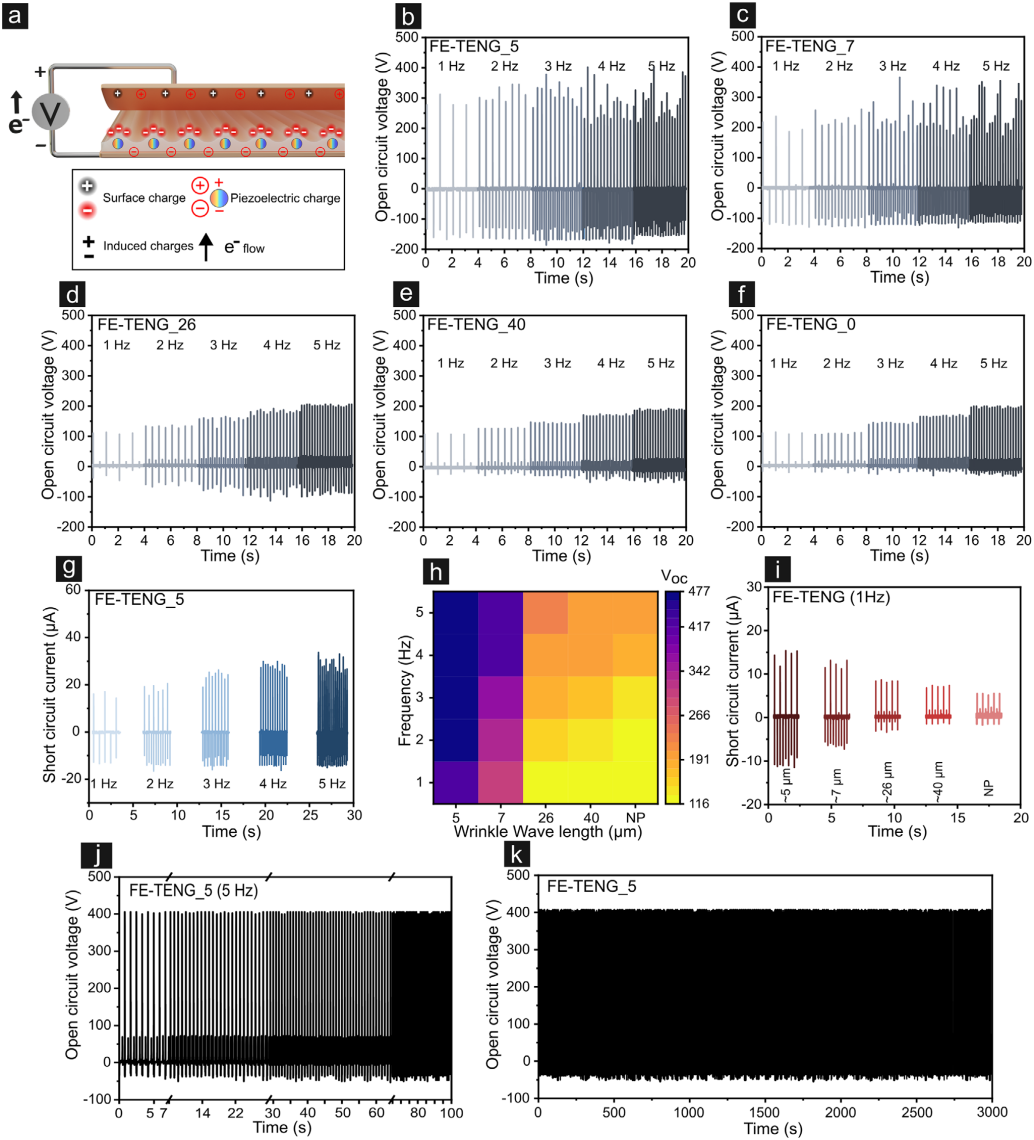
Frequency‐dependent electrical performance and stability of imprinted PVDF sensors. a) Schematic illustration of triboelectric and piezoelectric charge generation under mechanical deformation. b–f) Comparison of open‐circuit voltage (*V*_OC_) outputs measured across frequencies from 1 to 5 Hz for sensors featuring periodic surface structures of approximately 5, 7, 26, 40 µm and non‐patterned (FE‐TENG_0), respectively. g) Short‐circuit current output of the FE‐TENG_5 device at different frequencies, demonstrating consistent and stable current generation. h) Color mapping visualizing the combined influence of the imprinted PVDF's periodicity and vertical contact–separation mode frequency on the triboelectric V_OC_, providing insights into device optimization. i) Comparison of short‐circuit current at 1 Hz for various wrinkle periodicities and non‐patterned (NP) control, highlighting morphology‐dependent electrical output. j,k) Long‐term stability evaluation under repeated cyclic impacts (up to 3000 cycles) at an applied pressure of 3 N and a frequency of 5 Hz, confirming the sensor's robustness and reliability over extended use.

Detailed triboelectric analyses were carried out in our state‐of‐the‐art, pneumatically controlled, vertical contact‐separation motion‐driven triboelectric analyzer. The details of the instrument were elaborately described in our previous publications.^[^ ^72^ ^]^ For the measurements, we systematically evaluated the electrical signals (in terms of *V*_OC_ and *I*_SC_) of FE‐TENGs (with variable periodicity of wrinkles) for the contact area of (1.5 × 1.5) cm^2^ to validate the effectiveness of anisotropic TENG constructions designed for precise motion detection and ambient mechanical energy harvesting. The overall thickness of the FE‐TENG and effective friction layer are ≈16 µm and ≈1 µm, respectively. To ensure the scientific rigor of the experiment, we tested the single‐electrode FE‐TENG systems three consecutive times. **Figure** **3**b–f illustrates *V*_OC_ under the operating frequencies of 1–5 Hz and a friction separation distance of 10 mm, with a maximum *V*_OC_ of 420 V (5 Hz) reported for FE‐TENG_5, in contrast to its non‐wrinkled counterpart (NP, 190 V at 5 Hz). Throughout the testing process, a control variable method was employed for comparative experiments. As shown in Figure 3, the open‐circuit voltage (*V*_OC_) increases with the friction frequency (except for FE‐TENG_5 and FE‐TENG_7, in which the trends are less obvious) and decreases as the periodicity of the imprinted structure increases. The increment in electrical output is manifested by several key factors, including the surface contact area, the thickness of the active layer, operating frequency, humidity of the test compartment, extent of surface micropatterning/effective friction points etc.^[^ ^73^ ^]^

Evidently, FE‐TENG_5 demonstrates an overall output enhancement of more than 200% (5 Hz) from that of pristine, non‐wrinkled FE‐TENG_0. In conjunction with our previous reports and discussions in the preceding section, the superior output in FE‐TENGs owes primarily to the ferroelectric polarization of PVDF (piezoelectric effect), coupled with friction‐induced triboelectric charge generation. A hybridized mechanism inherently associated with the synergistic effect of the two results in superior charge density and output currents. Keeping in mind that the overall thickness of the FE‐TENGs remains unchanged (≈16 µm), a staggering 200% hike in output could only be associated with the higher effective friction contact points in FE‐TENGs with lower periodicity. Increased periodicity of the imprinted structures decreases the contact points with the top electrode, thereby resulting in reduced friction and hence, overall *V*_OC_. The frequency effect appears to be inconclusive in the case of FE‐TENG_5 and FE‐TENG_7, in which case, relative fluctuation in voltage waveforms is also noticeable. To explain this, understanding the hybrid piezoelectric‐triboelectric mechanism is crucial. For the piezoelectric effect, induced dipoles of ferroelectric polymer upon exposed to ac field orient themselves along the direction of field, giving rise to dielectric polarization. Higher piezo polarization would only be caused by higher effective polymer concentration. As the FE‐TENGs are constant in terms of layer thickness and area, the piezoelectric contribution would be approximately comparable to each other. However, triboelectricity is directly proportional to the friction contact points, therefore, contribution of triboelectric voltage would be maximal in these two, eventually driving the total hybridized output higher. A detailed comparison of piezoelectric and triboelectric output performance is provided in Figure S5 (Supporting Information). Moreover, as the wrinkled surface is irregular and inhomogeneous compared to pristine PVDF, the trend in voltammogram appears to be noisy, particularly at higher operating frequencies. However, the output trend in short circuit current (*I*_SC_, Figure 3g) for FE‐TENG_5 follows a better correlation with frequency, in which higher frequency results in improved overall output current. To compare and correlate the parameters affecting hybridized outputs, a 2D heat map was constructed (Figure 3h), illustrating the combined influence of operating frequency and wrinkle periodicity on the open‐circuit voltage. Furthermore, short‐circuit current (*I*_SC_) measurements acquired at a constant frequency of 1 Hz (Figure 3i) revealed that the device with 5 µm wrinkle periodicity (FE‐TENG_5) exhibits the highest current output, confirming that smaller periodicities yield improved triboelectric performance. The wrinkle morphology and geometrical dimensions, critical to charge generation, were extracted and visualized through SEM and schematic illustrations (Figure S7, Supporting Information). These parameters were subsequently used in FEM simulations to model the surface potential distribution. Notably, the hybrid electrical response is also governed by the crystalline structure of PVDF. FTIR and XRD analyses (Figure S8, Supporting Information) confirmed the presence of electroactive β‐ and γ‐phases in the imprinted PVDF, substantiating the material's intrinsic piezoelectric contribution.

Stability and durability are equally essential for practical TENG applications. The FE‐TENG_5 device was subjected to over 3000 contact‐separation cycles at 5 Hz with a separation distance of 10 mm. As shown in Figure 3j,k, the electrical output remained consistent, indicating excellent operational robustness. A comparable long‐term test under ambient tactile force (3 N) also demonstrated stable short‐circuit current output (Figures S9 and S10, Supporting Information).

### 2.4 Demonstration of Imprinted PVDF FE‐TENG in Sensing Applications

**Figure 4:**
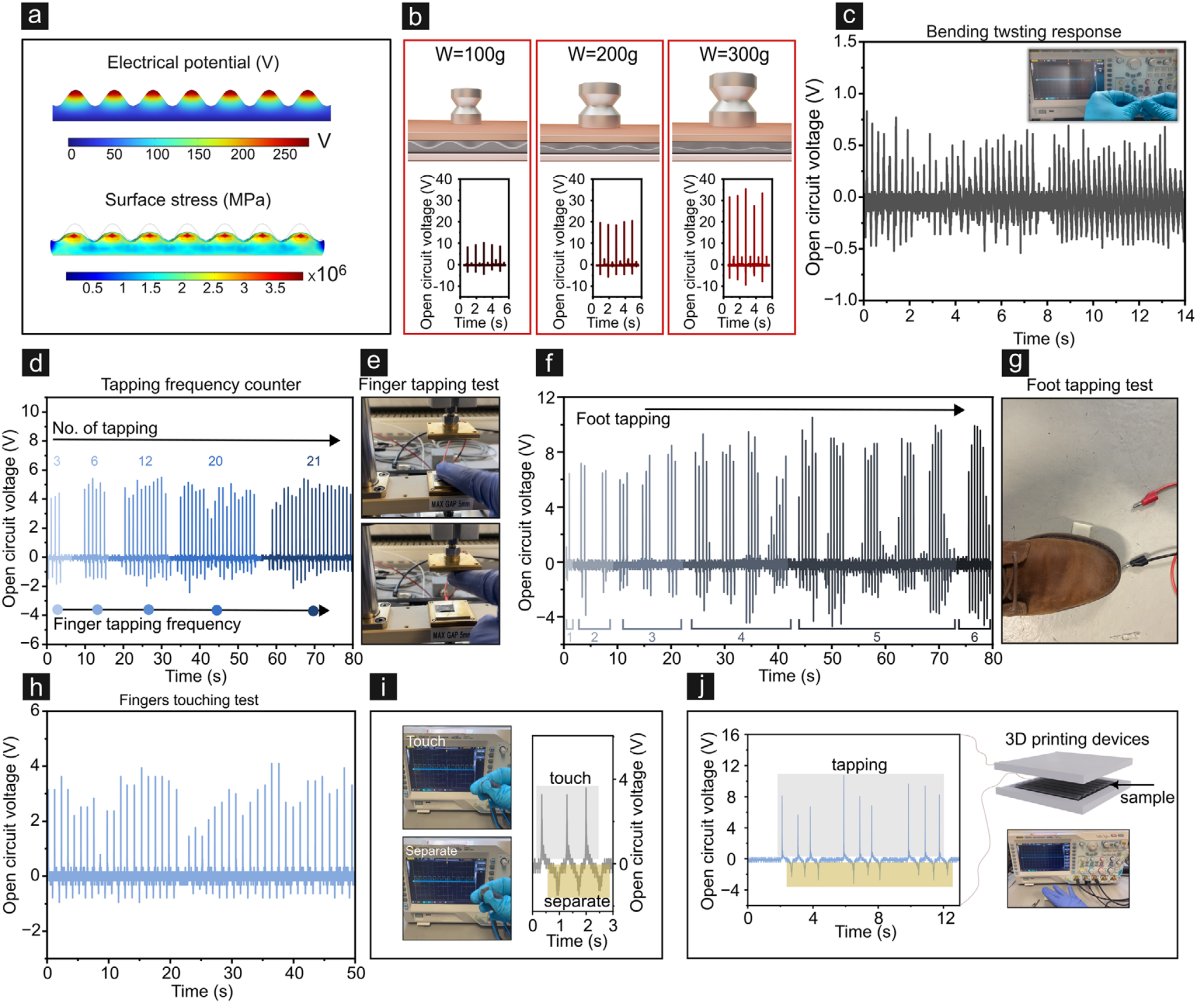
Ultra‐sensitive tactile sensing performance of the imprinted PVDF sensor: a) Finite element method (FEM)‐based numerical simulation illustrating the enhanced surface charge distribution and triboelectric potential of the microstructured sensor. b) Demonstration of the sensor's precise force detection capability using calibrated weights ranging from 100 to 300 g. c) Dynamic response of the device under simultaneous bending and twisting, illustrating robust output under complex mechanical deformations. d,e) Finger‐tapping frequency detection using the FE‐TENG_5 device, which can precisely count the number of taps based on the number of full waveforms, with a resolution of up to 10 Hz. Photographs show the actual test setup. f,g) Force sensing measurements under varying foot‐tapping frequencies, highlighting the corresponding *V*_OC_ responses and overall sensitivity of the sensor. h) Schematic representation of a 3D‐printed thermoplastic polyurethane (TPU) capsule used for controlled tactile testing, enabling consistent and reproducible sensor performance assessments.

The adaptability of the manufactured FE‐TENGs was evaluated by testing its performance under different separation distances between friction layers (Figure 3i). As the periodicity increases, the short‐circuit current decreases, following a trend similar to that observed in the open‐circuit voltage. The short‐circuit current of the FE‐TENG is measured at a constant frequency of 1 Hz for different surface wrinkle wavelengths (≈5 to ≈40 µm) and a non‐patterned (NP) surface (Figures 3i and S10, Supporting Information). The results demonstrate that structured surfaces enhance current output compared to the non‐patterned design, emphasizing the critical role of surface morphology in optimizing electrical performance. To validate these results, we simulated the electrical output of the imprinted PVDF using the finite element method (FEM) with COMSOL Multiphysics (**Figure** **4**a). In this simulation, the base film thickness is taken as 20 µm, and the microstructure distance is 10 µm, and a height of 5 µm, closely matching the experimental conditions. The wrinkle profile, including key structural parameters, is illustrated in Figure S7 (Supporting Information).

The simulated surface charge distribution of the imprinted PVDF structure is substantially higher than that of a flat PVDF film without surface structuring, such as wrinkles in this case. This result indicates a significant enhancement of the triboelectric potential, attributed to the increased effective surface area provided by the presence of the wrinkles.

To demonstrate the FE‐TENG_5′s excellent tactile and force sensing abilities, we investigated various wearable and force/pressure applications. As both triboelectricity and piezoelectricity are proportional to the friction force magnitude, force sensitivity experiments are important to validate the device's operability and adaptability. For that, we introduced a hybrid FE‐TENG sensor to detect and calibrate forces generated by dropped weights and finger‐tapping interactions, demonstrating its precision and versatility in diverse force‐sensing applications. Figure 4c showcases the sensor's excellent performance by demonstrating its response to different weighted objects, ranging from a 100 g alumina load to weights between 200 g and 300 g. The resulting output waveform displays three distinct triboelectric peaks corresponding to the applied weights. The device also maintains stable electrical output under complex deformations such as bending and twisting (Figure 4c), showcasing excellent mechanical flexibility. In Figure 4d–g, evaluates the device's performance under finger and foot tapping conditions across varying frequencies, revealing a direct correlation between tapping frequency and voltage output (Video S1, Supporting Information). This highlights the device's dynamic responsiveness to mechanical forces. Figure 4e depicts the experimental setup used to simulate practical finger‐tapping scenarios, confirming the device's functionality and reliability in real‐world applications.

Considering the excellent sensitivity and output performance of the imprinted PVDF‐based FE‐TENG_5, this design has been applied to harness mechanical energy generated by finger‐tapping motions and to employ it in frequency counter devices. As shown in Figure 4d (Figure S9 and Video S2, Supporting Information) tapping a human finger on the sensor generated an open‐circuit voltage (*V*_OC_) of 6 V. This experiment demonstrates the applicability of the device for quantitative estimation of tap or stroke (up to 10 Hz resolution) for potential deployment as frequency counter. Each tap is represented by a full‐cycle waveform in the voltammogram, which gives rise to a higher number of peaks with increased tapping. The amplitude is proportional to tapping force and doesn't deviate significantly when force is maintained below 10 N. This feature will enable future integration in electronic switch and frequency counter devices. Furthermore, the functionality is further extended to foot tapping mode of operation. When the TENG is placed under a shoe sole to collect mechanical energy from human footsteps, a *V*_OC_ of 10 V is obtained. These sensors demonstrate an exceptional ability to detect low‐pressure and low‐intensity tactile responses when attached to the index fingertip of a human hand (refer to the inset in Figure 4i). This intuitive correlation between output voltage and mechanical stimulus is of significance for effective self‐powered sensing. Similar methodologies were adopted by Lin et al., who designed a gear‐driven triboelectric sensor that could simultaneously capture rotational speed, displacement and acceleration with high sensitivity, thereby eliminating the need for complex signal transformation techniques.^[^ ^74^ ^]^ It exhibits consistent and accurate tactile pressure detection, allowing for precise tracking of finger movements during frequent contacts. Additionally, these sensors possess high‐pressure sensitivity, enabling the detection of even subtle finger movements. Figure 4h–j demonstrates the high sensitivity of the sensor to finger and thumb touching, producing stable and precise output responses. The imprinted PVDF TENG exhibits excellent sensitivity, making it suitable for monitoring finger‐touch inputs. A comparable long‐term test under ambient tactile force (3 N) also demonstrated stable short‐circuit current output (Figures S9 and S10, Supporting Information). It is also worth noting that the output power density reported for FE‐TENGs appeared higher or comparable to that of recently reported hybrid systems (Figure S11 and Table S5, Supporting Information). These results indicate that the wrinkling‐based imprinted PVDF‐based TENGs can be effectively used as a generator for harvesting mechanical energy from human activities (Figure S9 and Video S3, Supporting Information). This demonstrates its potential application in micro‐wearable devices, providing highly sensitive sensors for microwearable electronics.

## 3 Conclusion

In summary, we have developed a novel PVDF‐based, wrinkled FE‐TENGs integrated with a modular tactile sensor through a lithography‐free fabrication approach. By coupling wrinkling and spin‐coating methodologies, this scalable and reproducible approach enabled the generation of microstructured surfaces on PVDF substrates while preserving the structural integrity of the wrinkles. The wrinkling technique, induced onto PDMS provided a cost‐effective, energy efficient alternative to conventional top‐down patterning method. Our approach facilitated the fabrication of high‐performance FE‐TENG devices, achieving a hybridized electrical output approximately 200% higher than pristine PVDF substrates. A maximum power density of 0.9 mW cm^−^^2^ was obtained at the smallest wrinkle periodicity (≈5 µm), highlighting the critical role of microstructure geometry on device efficiency. Conversely, increased periodicity resulted in diminished output due to reduced frictional contact density. Notably, the hybrid device exhibited exceptional force‐sensing capabilities, with the ability to detect forces as low as 2 N, thereby making it a highly promising candidate for tactile sensing applications. These high sensitivity microstructured hybrid sensors combine superior performance, ease of integration, and intrinsic self‐powered operation, aligning with recent advancements in multifunctional TENG‐based sensors designed for dynamic mechanical monitoring applications. Their outstanding tactile resolution and force‐sensing capabilities underscore their potential in diverse applications, including healthcare data collection, robotic systems, and artificial intelligence. To our best knowledge, this is the first demonstration of an efficient, lithography‐free strategy for producing high‐performance microstructured FE‐TENGs using programmable wrinkling. The synergistic hybrid mechanism enhances electrical output under cyclic contact‐separation motions, enabling effective harvesting of ambient mechanical energy. This work lays a strong foundation for the future development of advanced tactile sensors, particularly in applications requiring precise force detection and high‐resolution tactile sensing. Beyond conventional domains, the inherent biocompatibility of PVDF opens exciting opportunities in tissue engineering and other biologically integrated technologies. Wrinkled PVDF microstructures may further improve cell adhesion, mechanical compatibility, and sensory interfacing in biological environments, paving the way for innovative applications in bio‐integrated sensing and actuation.

## 4 Experimental Section

### PDMS Wrinkle Formation

Polydimethylsiloxane (PDMS) sheets, each 2 mm thick, were fabricated using the Sylgard 184 kit (Dow Corning, USA). The material was prepared by mixing the dimethylsiloxane oligomer and platinum‐based crosslinking agent in a 10:1 weight ratio. To generate a variety of wrinkle patterns and periodicities, two distinct surface treatment techniques were utilized: low‐pressure plasma treatment for periodicities ranging from 1 to 8 µm and ultraviolet/ozone (UV/O₃) treatment for larger periodicities between 20 µm and 50 µm. The PDMS curing procedures were customized according to the requirements of each treatment method. Samples subjected to low‐pressure plasma treatment were cured at room temperature for 48 h. For UV/O₃ treatment, a two‐step curing protocol was applied, comprising an initial curing phase of 48 hours at room temperature, followed by an additional 4 hours at 80 °C. To ensure consistent thickness, the curing process was performed on a leveled surface. Once cured, the PDMS sheets were cut into strips and mounted on a custom stretching device for further processing.

For the low‐pressure plasma treatment, a MicroSys system (Roth & Rau, Wüstenbrand, Germany) was employed. The system featured a stainless‐steel vacuum chamber (350 mm in diameter and height), which was evacuated to a base pressure below 10⁻⁷ mbar using a turbomolecular pump. Plasma was generated using a 2.46 GHz electron cyclotron resonance source (RR160 by Roth & Rau) with a 160 mm diameter and a maximum power output of 800 W. Samples were introduced into the chamber via a load‐lock system and placed centrally on a fixed holder approximately 200 mm from the plasma excitation zone. Hydrogen gas (purity 99.999%, Air Liquide) was supplied through a controlled flow system, maintaining a process pressure of 2 × 10⁻^2^ mbar. PDMS strips measuring 45 × 20 × 2 mm^3^ were stretched to 130% of their original length and exposed to H₂ plasma for either 140 seconds or 380 seconds at 800 W, depending on the desired treatment. For UV/O₃ treatment, a Novascan PSD‐UV8 system (Novascan Technologies, Ames, USA) operating at ambient pressure was used. Stretched PDMS samples (100 mm × 50 mm × 2 mm), extended to 170% of their original length, were positioned at a fixed distance from the UV source and exposed for 45 minutes or 120 minutes, depending on the target wrinkle periodicity.

### Imprinted PVDF Sensor Formation

A 20 wt% PVDF solution was prepared by dissolving PVDF pellets (Solef 6008/0001, Germany) in 99.5% N,N‐dimethylformamide (DMF) under continuous stirring to ensure complete dissolution. This solution was then spin‐coated onto microstructured, wrinkled PDMS templates at a speed of 1000 rpm for 60 s, resulting in a uniform coating layer. Following the spin‐coating process, the coated film was dried at 80 °C for 30 min to remove residual solvent. To enhance the crystalline structure and promote the formation of the β‐phase of PVDF, the film underwent annealing at 150 °C for 2 h. After annealing, the film was gradually cooled to room temperature to mitigate thermal stresses and preserve the integrity of the wrinkled microstructure. The resulting wrinkled PVDF film was subsequently used for electrical characterization. For the fabrication of freestanding triboelectric devices, the films were cut into 1.5 cm × 1.5 cm squares, ensuring consistency in size for subsequent experimental evaluations.

### Scanning Electron Microscopy

High‐resolution scanning electron microscopy (SEM) images were acquired using a NEON 40 FIB‐SEM workstation (Carl Zeiss Microscopy GmbH, Oberkochen, Germany) operating at an electron high tension (EHT) of 3.5 kV. To improve surface conductivity and achieve optimal imaging quality, the samples were coated with a 3 nm layer of platinum using a sputter coater (Leica Microsystems GmbH, Wetzlar, Germany). Imaging was conducted with the secondary electron (SE2) detector for enhanced surface detail visualization.

### Confocal Microscopy

3D surface imaging was conducted using a spinning disk confocal microscope (µSurf expert, Nanofocus AG, Oberhausen, Germany). This advanced system provides non‐contact 3D profiling with vertical resolution at the sub‐micrometer scale, making it particularly well‐suited for quantitative analysis of surface topography. It was employed to accurately measure key characteristics such as the amplitude and periodicity of the wrinkled structures on PDMS and PVDF surfaces.

### Triboelectric Measurement

The open‐circuit current and voltage characterization of the microstructured PVDF sensor was performed using a custom‐designed vertical contact‐separation system driven by a pneumatic actuator (Festo Corp.). The pneumatic motor, controlled by compressed air pressure, can generate a maximum vertical contact force of 500 ± 20 N. For experiments involving the wrinkled PVDF sensor, the contact force was maintained at a consistent 200 ± 20 N (corresponding to 3 bar compressed air pressure), unless otherwise specified. Measurements were recorded using a 3‐channel oscilloscope, where the channels were assigned to monitor the trigger voltage, open‐circuit voltage output, and applied force in newtons, respectively. A Rigol DS4024 Digital Storage Oscilloscope was used, interfaced with a custom LabVIEW program through an NI‐9239 measurement system connected via a USB carrier. This setup enabled real‐time monitoring and synchronization of the force, trigger voltage, and output voltage or current waveforms. For energy harvesting assessments, the wrinkled PVDF sensors were connected to a full‐wave bridge rectifier circuit incorporating ceramic resistors of varying values (spanning from 1 kΩ to 47 MΩ). This configuration allowed evaluation of the sensors' performance across different load conditions.

### Statistical Analysis

All electrical measurements, including open‐circuit voltage (*V*_OC_), short‐circuit current (*I*_SC_), and calculated power density, were repeated at least three times to ensure consistency and reproducibility. The reported values represent the mean ± standard deviation (SD) unless otherwise stated. For each device configuration (e.g., wrinkle periodicities), data was collected under identical experimental conditions. Time‐resolved electrical data was acquired using a 3‐channel digital oscilloscope connected to a custom LabVIEW interface, with real‐time synchronization of applied force and signal response and resulting data were analyzed and processed using OriginPro 2023. For transferred charge estimation, short‐circuit current data were numerically integrated over time using the Simpson's rule, and the result was reported in µC. Data analysis was performed exclusively using OriginPro 2023 and MS Office Excel.

## Conflict of Interest

The authors declare no conflict of interest.

## Supporting information



Supporting Information

Supplemental Video 1

Supplemental Video 2

Supplemental Video 3

## Data Availability

The data that support the findings of this study are available from the corresponding author upon reasonable request.
