# Supplementary material for: Transfer‐Printed Wrinkled PVDF‐Based Tactile Sensor‐Nanogenerator Bundle for Hybrid Piezoelectric‐Triboelectric Potential Generation
Source: Small. 2025 May 8;21(26):2502767. doi: 10.1002/smll.202502767 (PMC12232232; doi:10.1002/smll.202502767)
Supplement: Supplementary file 1 — Supporting Information [file SMLL-21-2502767-s002.pdf]

NANO · MICRO
small

Supporting Information

for *Small*, DOI 10.1002/smll.202502767

Transfer-Printed Wrinkled PVDF-Based Tactile Sensor-Nanogenerator Bundle for Hybrid Piezoelectric-Triboelectric Potential Generation

Kamal Kumar Meena, Injamamul Arief, Anik Kumar Ghosh, André Knapp, Mirko Nitschke, Andreas Fery and Amit Das*

Supporting Information

Transfer-Printed Wrinkled PVDF-Based Tactile Sensor-Nanogenerator Bundle for Hybrid Piezoelectric-Triboelectric Potential Generation

Kamal Kumar Meena,[1,‡] *Injamamul Arief,*[1,‡,*] *Anik Kumar Ghosh,*[1,‡] *André Knapp,*[1] *Mirko Nitschke,*[1] *Andreas Fery*[1,2] *and Amit Das*[1,3]

[1]Leibniz-Institut für Polymerforschung Dresden e.V., Hohe Str. 6, D-01069 Dresden, Germany

[2]Center for Advancing Electronics Dresden (cfaed), Technische Universität Dresden, Helmholtzstr. 18, 01069 Dresden, Germany

[3]Tampere University, 33720 Tampere, Finland

[‡]These authors contributed equally
[*]Corresponding author: arief@ipfdd.de

Wrinkle Template Fabrication and Visualization

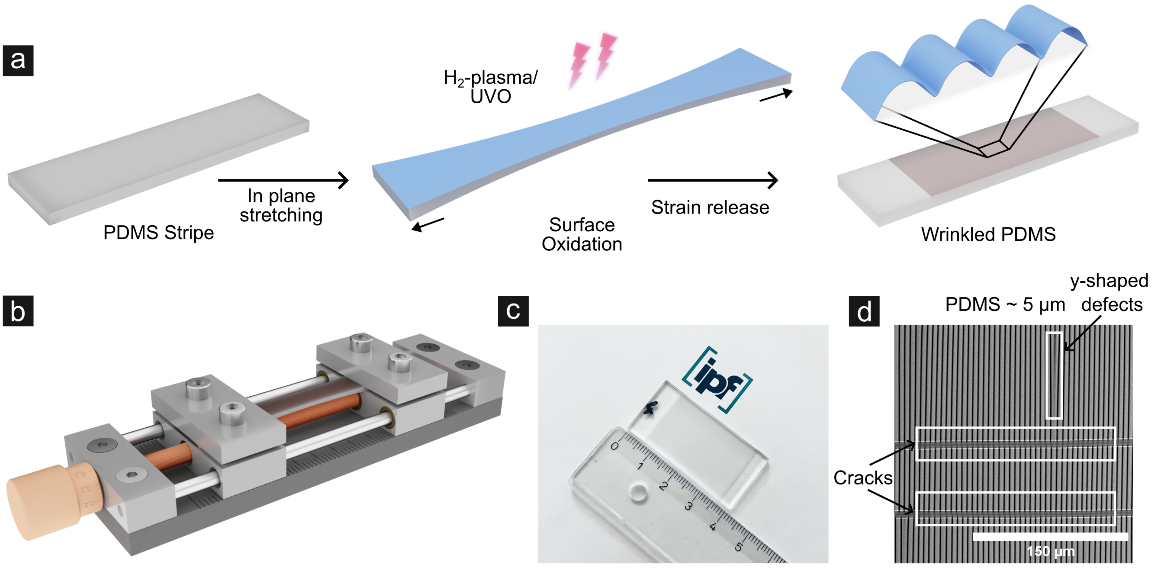

Figure S1. (a) From left to right: A PDMS substrate of specified dimensions (left), undergoing in-plane stretching and oxidative treatment (middle), and subsequent strain release resulting in wrinkle formation (right). (b) A custom-built stretching device designed for in-situ oxidation of PDMS substrates. (c) A digital photograph of the wrinkled PDMS strip. (d) A SEM image of the wrinkled PDMS surface (periodicity $\sim 5\,\mu m$).

Figure S1 demonstrates the process of creating wrinkled PDMS substrates using custom-made stretching device specifically designed for in situ treatments. Figure S1a illustrates the uniaxial in-plane stretching of the PDMS substrate, surface modification in the presence of oxidative UV/O_3 medium & H_2-plasma, followed by strain release procedure. Figure S1b depicts the custom-made stretching device, which applies precise strain to the PDMS stripe. This device features an adjustable mechanism to ensure uniform elongation of the sample, a critical factor for achieving consistent surface patterns. A digital photograph of the actual wrinkled PDMS template prepared during the process is shown in Figure S1c, emphasizing the practical realization of the fabrication steps. Finally, Figure S1d presents an SEM image of the wrinkled PDMS template, confirming the formation of microstructuring with a periodicity of $\sim 5\,\mu m$.

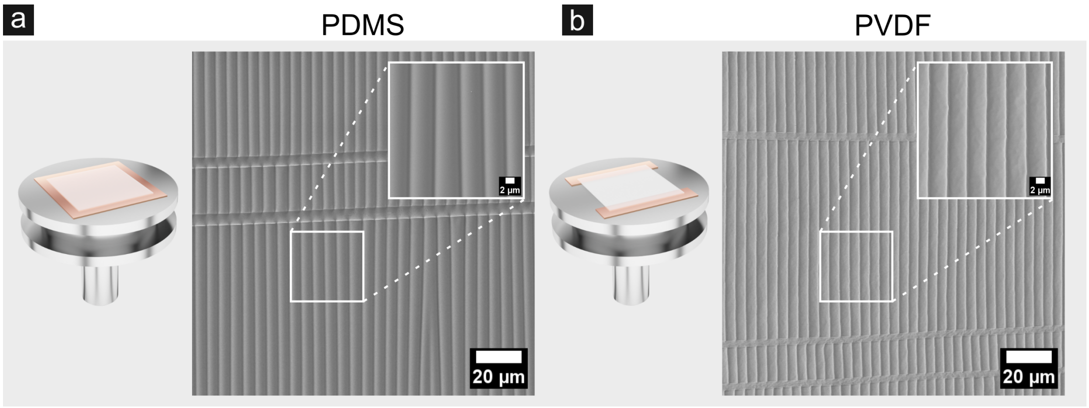

Figure S2. (a) Mounting of the wrinkled PDMS template (with a periodicity of ~ 5 μm), along with its corresponding SEM image. (b) Mounting of the imprinted PVDF film, accompanied by SEM images (inset scale bars: 2 μm for both cases).

Different mounting procedures were followed for the wrinkled PDMS template and the imprinted PVDF substrate. For the moderately thick wrinkled PDMS template, the adhesive layer fully covers the substrate (as shown in Figure S2a). In contrast, for the free-standing imprinted PVDF, which is much thinner, the adhesive layers are positioned to leave a gap between them (as shown in Figure S2b). This process was followed to prevent any local distortion of the imprinted PVDF film while imaging.

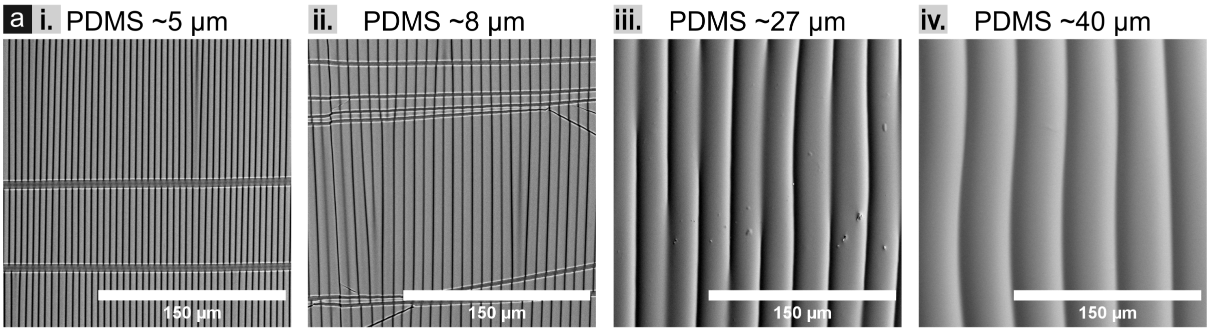

Figure S3. (a) SEM images of the wrinkled PDMS template with periodicities of ~ 5 μm (i), ~ 8 μm (ii), ~ 27 μm (iii), and ~ 40 μm (iv), respectively. The exact values of these periodicities are listed in Table S1.

Table S1. Calculated periodicities of the wrinkled PDMS template and the imprinted PVDF replica. Notably, these values were derived by averaging the data across the entire topography.

Sample	Periodicity (µm)	Amplitude (µm)
PDMS (~ 5 µm)	4.68±0.11	1.46±0.03
PVDF (~ 5 µm)	4.72±0.66	0.50±0.12
PDMS (~ 8 µm)	7.88±0.32	2.46±0.08
PVDF (~ 7 µm)	7.45±0.63	1.48±0.26
PDMS (~ 27 µm)	27.16±2.30	3.37±0.78
PVDF (~ 26 µm)	26.48±2.93	2.10±0.61
PDMS (~ 40 µm)	39.86±5.77	10.22±1.77
PVDF (~ 40 µm)	40.07±5.30	6.16±1.44

Table S2. Calculated periodicities of the wrinkled PDMS template and the imprinted PVDF replica: These values were derived by averaging measurements from four randomly selected peaks of a line profile.

Sample	Periodicity (µm)	Amplitude (µm)
PDMS (~ 5 µm)	4.61±0.04	1.39±0.11
PVDF (~ 5 µm)	4.85±0.13	0.90±0.03
PDMS (~ 8 µm)	8.20±0.14	2.56±0.04
PVDF (~ 7 µm)	7.15±0.20	1.71±0.06
PDMS (~ 27 µm)	26.47±2.84	3.66±0.49
PVDF (~ 26 µm)	28.64±0.73	6.38±0.67
PDMS (~ 40 µm)	44.07±3.30	11.26±0.30
PVDF (~ 40 µm)	39.53±1.33	7.95±0.43

Table S3. Amplitude and periodicity ratio calculations. The values of imprinted PVDF are in the numerator, while the values of wrinkled PDMS are in the denominator. Approximate periodicities of imprinted PVDF are listed in the leftmost column, with the exact values provided in Table S1. Exact values enlisted in Table S1 are used for this calculation.

Periodicity (µm)	Amplitude Ratio† (µm)	Periodicity Ratio‡ (µm)
~ 5	0.65	1.05
~ 7	0.67	0.87
~ 26	1.74	1.08
~ 40	0.71	0.90

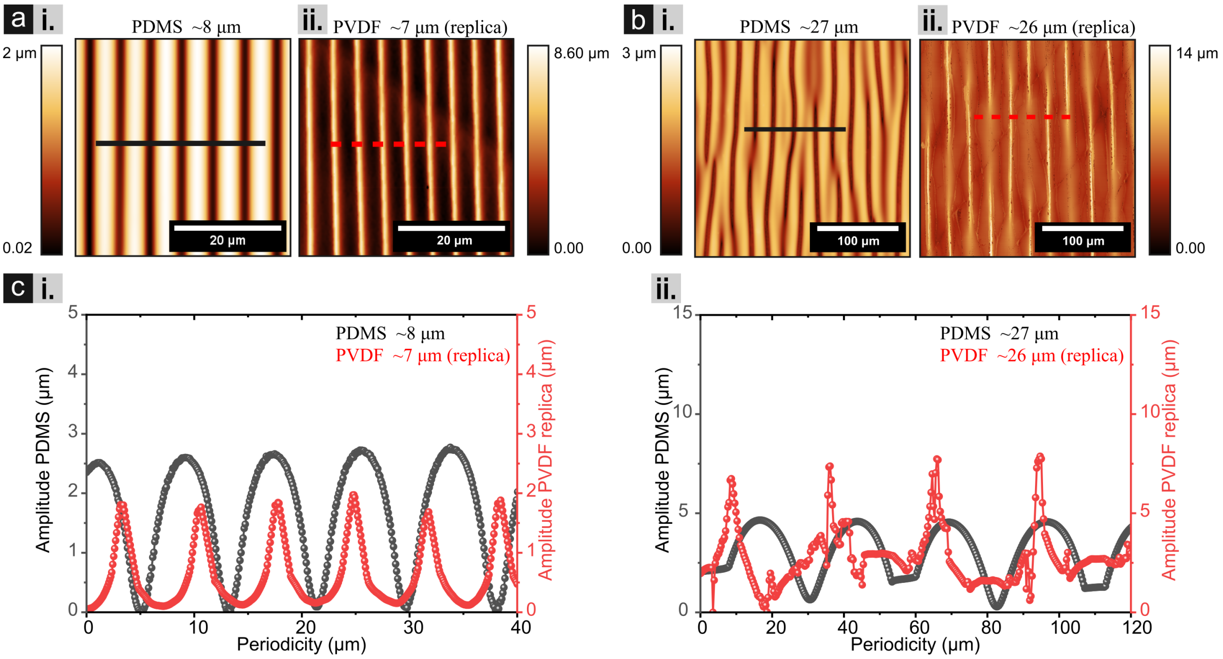

Figure S4. (a) Confocal microscopic images of the wrinkled PDMS template and the corresponding imprinted PVDF patterns, showing periodicities of 7.88 ± 0.32 μm (i) and 7.45 ± 0.63 μm (ii), as well as 27.16 ± 2.30 μm for PDMS (b-i) and 24.72 ± 4.97 μm for PVDF (b-ii), respectively. Scale bars: 20 μm in (a) and 100 μm in (b). (c) Line profile comparisons between the wrinkled PDMS template and the imprinted PVDF structures, highlighting periodicities ~ 8 μm (~ 7 μm for PVDF) (i) and ~ 27 μm (ii) (~ 26 μm for PVDF). The locations of the extracted profiles are indicated in (a) and (b).

Coefficient of Variation (CV) and Accuracy Calculation in Percent

The reliability and consistency of the imprinting process were quantitatively evaluated using the coefficient of variation (CV) and accuracy (%). The CV, obtained by normalizing the standard deviation against the mean, measures the relative variability of the imprinted structures, thereby indicating their uniformity. Accuracy evaluates how closely the observed imprinted structures match the ideal ones, thus serving as a direct metric of the imprinting precision. Together, these measures provide a comprehensive framework for assessing reproducibility and reliability, ultimately guiding targeted improvements to achieve high-fidelity microstructure fabrication.

$$\text{CV}(\%) = \left(\frac{\text{Standard Deviation (SD)}}{\text{Mean Ratio}}\right) \times 100 \tag{S1}$$

$$\text{Accuracy}(\%) = \left(1 - \left|1 - \frac{\text{PVDF Value}}{\text{PDMS Value}}\right|\right) \times 100 \tag{S2}$$

Table S4. Calculated values of CV and accuracy (in %) for the imprinted PVDF substrate. The values presented in Table S2 were used to determine the values listed here.

Periodicity (µm)	Amplitude CV(%)	Amplitude Accuracy(%)	Periodicity CV(%)	Periodicity Accuracy(%)
~5	3.33	64.75	2.68	94.79
~7	3.51	66.80	2.80	87.20
~26	10.50	25.68	2.55	91.80
~40	5.41	70.60	3.36	89.70

Comparison of Piezoelectric and Triboelectric output Performance

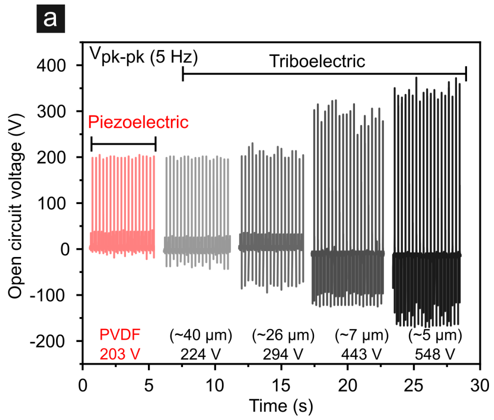

Figure S5. Comparison of piezoelectric and triboelectric output performance. (a) Piezoelectric and triboelectric output performance comparisons for devices without microstructure films and with different wrinkle wavelengths (~ 5, ~ 7, ~ 26, and ~ 40 µm) under 3 N pressure.

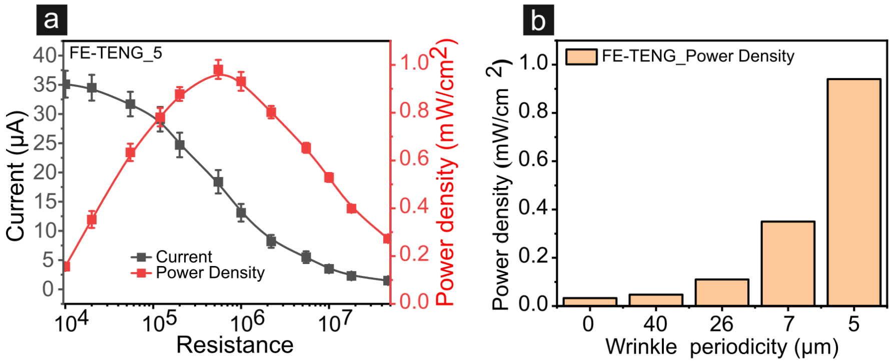

Figure S6. (a) Comparison of power density between pristine and wrinkled FE-TENG_5 devices. (b) Histogram displaying the distribution of power densities for various FE-TENG configurations.

FEM Modeling

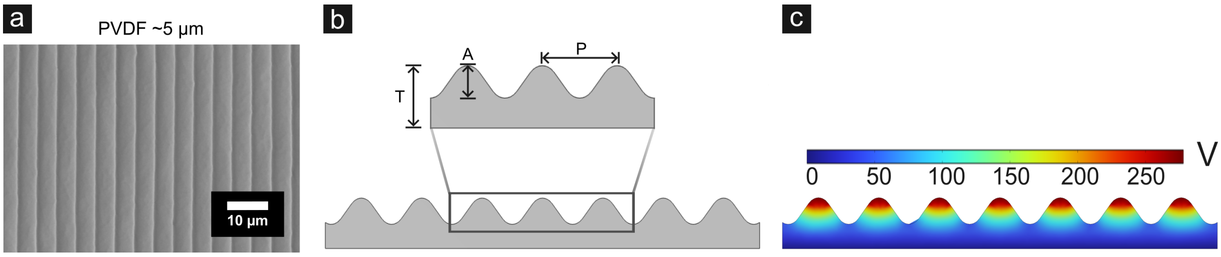

Figure S7. (a) SEM image of the fabricated surface, highlighting the imprinted PVDF structure with a periodicity of approximately 5 µm. (b) Schematic representation of the wrinkle profile, indicating key parameters: wrinkle amplitude (A), period (P), and thickness (T). (c) Simulated electrical potential distribution across the wrinkled surface (FE-TENG_5), illustrating how the potential (V) varies along the structure.

XRD-FTIR

Figure S8 shows the FTIR spectrum of spin-coated microstructures of PVDF film. The neat PVDF film primarily consists of α, β, and γ-crystalline phases, as evidenced by characteristic absorption peaks observed at 764 cm^{-1}, 975 cm^{-1} (α-phase) and 840 cm^{-1} 1275 cm^{-1}(β-phase), 840 cm^{-1} 1234 cm^{-1} (γ-phase). Additionally, the spectrum exhibits a peak at 840 cm^{-1}, indicating the presence of the β-phase (TTTT). The α-phase (non-electroactive) conversion to the γ-phase (semi-electroactive) was observed, showing vibrational bands at 764 cm^{-1}, 840 cm^{-1} and 1234 cm^{-1} with the peak at 1234 cm^{-1} uniquely indicative of the γ-phase.[1,2] The results indicate that a very the dipole moment of PVDF (–CH_2–/–CF_2–) and induce the formation of the semi-polar γ-phase (TTTGTTTG), which can be utilized for energy storage, the relative proportion of the electroactive phase (EA) in the PVDF micropatterns was determined using the following equation:

$$F_{EA} = \frac{A_{\beta,\gamma}}{\frac{K_{\beta,\gamma}}{K_\alpha}A_\alpha + A_\gamma} \quad \text{(S3)}$$

Where A_α and $A_{\beta,\gamma}$ are the absorbance at the wave numbers of 764 cm^{-1} and 840 cm^{-1} and K_α and $K_{\beta,\gamma}$ are the corresponding absorbance coefficients, 6.1 x $10^4 cm^2 mol^{-1}$ and 7.7 x $10^4 cm^2 mol^{-1}$, respectively. The X-ray diffraction (XRD) analysis confirmed the presence of different crystalline phases in the PVDF spin-coated film.

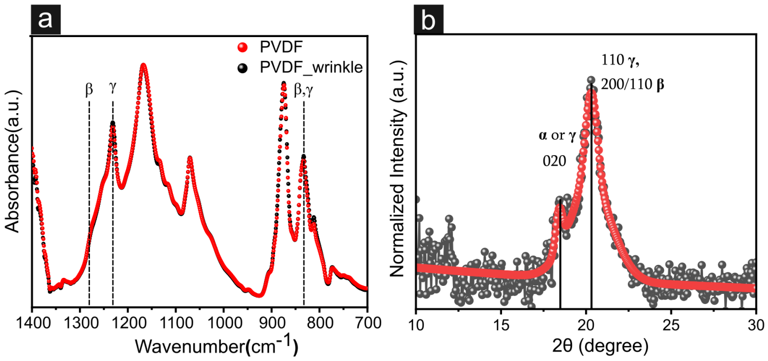

Figure S8. (a) FT-IR spectra of spin-coated pure PVDF and microstructures 5 μm. (b) X-ray diffraction patterns spin-coated pure PVDF and microstructures.

Figure S8 displays the XRD pattern of the microstructured PVDF film spectra. The XRD peaks observed in the pattern correspond to distinct crystal phases of PVDF. The peaks at 18.30° and 19.90° are attributed to the α-crystals, specifically the (020) and (110) planes, respectively. The peaks at 20.3° indicate the presence of β-crystals, corresponding to the (110) and (220) planes.[3] [4]Furthermore, the peaks at 19.9° and 20.04° are associated with the γ-phase, specifically the (002) and (110) planes, respectively.

Notably, the peak observed at approximately 20.30° in the PVDF film could be related to the γ or β-phase. Distinguishing between these phases based solely on XRD analysis can be challenging due to their proximity.

Application

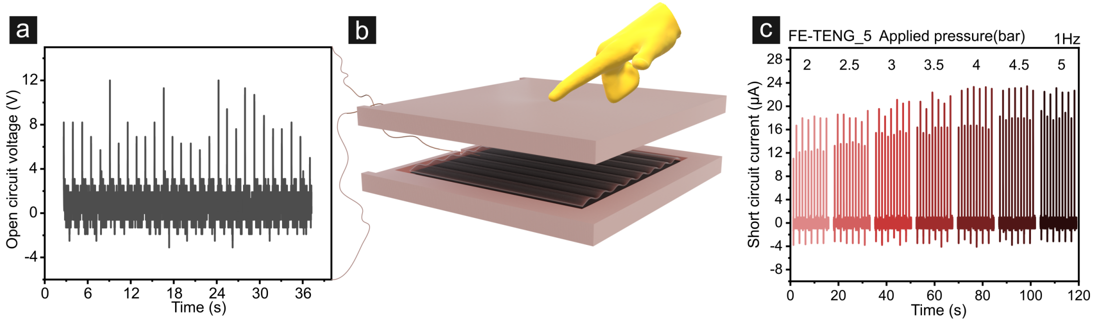

Figure S9. Energy harvesting under finger tapping: (a) Measured open-circuit voltage response during finger tapping. (b) The schematic of the circuit diagram of an energy harvester under finger tapping. (c) Short-circuit current (I_{SC}) output under varying applied pressures at 1 Hz frequency, demonstrating the device's ability to respond to increased mechanical stress As shown in Fig. 6a, the energy harvester generates an open-circuit voltage of about 12 V when the finger taps produce a relatively small pressure.

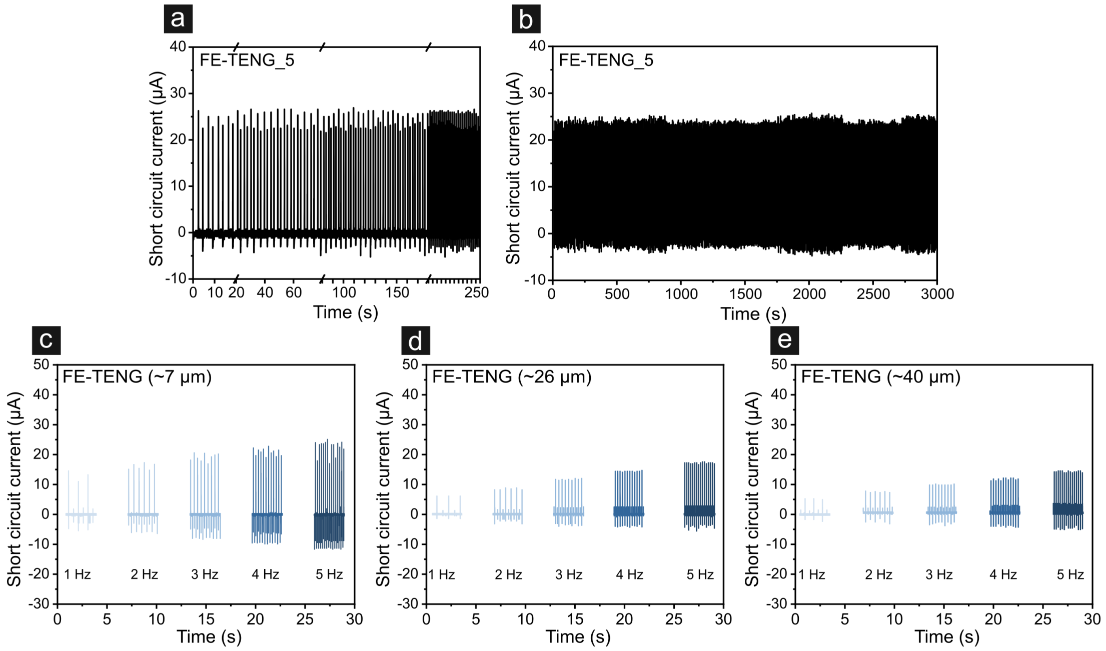

Figure S10. (a,b) Long-term durability test of the flexible hybrid nanogenerator (FE-TENG_5) showed stable short-circuit current (I_{SC}) output over 3000 cycles, confirming its stability and durability for long-term operation. (c-e) Frequency-dependent short-circuit current (I_{SC}) responses of FE-TENG devices with different wrinkle periodicities of ~ 7 µm, ~ 26 µm and ~ 40 µm, respectively.

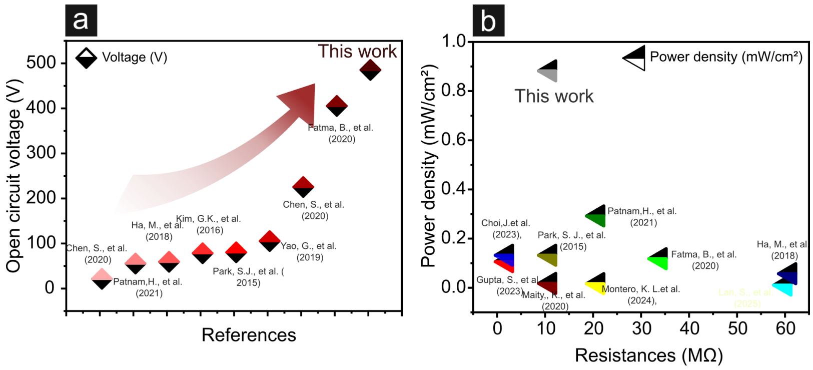

Figure S11. Comparative analysis of (a) open-circuit voltage and (b) power density of the current PVDF-based TENG versus previously reported studies.

Table S5. A relative comparison of the performance of PVDF-based microstructured pressure sensors and employing other materials too, as reported in recent years.

Materials/ Structure	Sensing range	Current (μA)	Voltage (V)	Power density ($\mu W/cm^2$)	Device Thickness (μm)	Device size (cm^2)	App.
PVDF/ Microstructure wrinkle[This work]	2-6N	31	~ 420	940	~ 16	2.25	Touch sensor and Pressure sensor
P(VDF-TrFE)/ PDMS Interlocked Microridge structure[5]	0-19.6 kPa	2.78	~ 60	46.7	~ 400	2.25	Wearable devices
PVDF / PVA Microstructure[6]	–	6	230	310	~ 50	4	Electronic devices
Graphene/ Ecoflex[7]	5 Pa - 20 kPa	0.3	–	–	~ 50	4	Touch sensor
Interlocked PVDF-TrFE Microstructure[8]	0.1-98 kPa	2.78	~ 85	–	~ 250	4	Thermal sensor
PDMS/Au NW Nanoflower structure[9]	5–50 kPa	5.5	~ 110	150	–	3.6	–
PDMS/ Microstructure[10]	5–50 kPa	0.262	3.14	–	> 550	1	Robotic Tactile Sensing
PDMS/ Hierarchically microstructure[11]	0.1-60 kPa	–	~ 26	23	~ 50	4.5	Pressure sensor
PEDOT:PSS/ Wrinkles and micro-cracks[12]	0 - 24 N	–	20.5	–	100	1	Tensile strain and Pressure sensor
Electrospun PVP/PVDF fiber[13]	0.2-2 kPa	~ 1.4	~ 6	–	~ 350	16	Pressure sensor
Al/Kapton linear micro-structures[14]	<294 kPa	44.5	560	62.3	100	400	–

References

[1] P. Martins, A. Lopes, S. Lanceros-Mendez, Electroactive phases of poly(vinylidene fluoride): Determination, processing and applications, Progress in Polymer Science 39 (2014) 683–706. doi:10.1016/j.progpolymsci.2013.07.006.

[2] H. Liu, Z. Wang, J. Xie, C. Guo, W. Hu, Control over the complex phase evolutions for ultrahigh dielectric energy storage in pure poly(vinylidene fluoride) films, Journal of Energy Storage 55 (2022) 105618. doi:10.1016/j.est.2022.105618.

[3] B. S. Ince-Gunduz, R. Alpern, D. Amare, J. Crawford, B. Dolan, S. Jones, R. Kobylarz, M. Reveley, P. Cebe, Impact of nanosilicates on poly(vinylidene fluoride) crystal polymorphism: Part 1. melt-crystallization at high supercooling, Polymer 51 (2010) 1485–1493. doi:10.1016/j.polymer.2010.01.011.

[4] X. Cai, T. Lei, D. Sun, L. Lin, A critical analysis of the α, β, and γ phases in poly(vinylidene fluoride) using ftir, RSC Advances 7 (2017) 15382–15389. doi:10.1039/c7ra01267e.

[5] M. Ha, S. Lim, S. Cho, Y. Lee, S. Na, C. Baig, H. Ko, Skin-inspired hierarchical polymer architectures with gradient stiffness for spacer-free, ultrathin, and highly sensitive triboelectric sensors, ACS Nano 12 (2018) 3964–3974. doi:10.1021/acsnano.8b01557.

[6] H. Patnam, B. Dudem, S. A. Graham, J. S. Yu, High-performance and robust triboelectric nanogenerators based on optimal microstructured poly(vinyl alcohol) and poly(vinylidene fluoride) polymers for self-powered electronic applications, Energy 223 (2021) 120031. doi:10.1016/j.energy.2021.120031.

[7] S. Chen, Y. Wang, L. Yang, F. Karouta, K. Sun, Electron-induced perpendicular graphene sheets embedded porous carbon film for flexible touch sensors, Nano-Micro Letters 12 (2020). doi:10.1007/s40820-020-00480-8.

[8] Y. Shin, Y. Park, S. K. Ghosh, Y. Lee, J. Park, H. Ko, Ultrasensitive multimodal tactile sensors with skin-inspired microstructures through localized ferroelectric polarization, Advanced Science 9 (2022). doi:10.1002/advs.202105423.

[9] S.-J. Park, M.-L. Seol, S.-B. Jeon, D. Kim, D. Lee, Y.-K. Choi, Surface engineering of triboelectric nanogenerator with an electrodeposited gold nanoflower structure, Scientific Reports 5 (2015). doi:10.1038/srep13866.

[10] G. Yao, L. Xu, X. Cheng, Y. Li, X. Huang, W. Guo, S. Liu, Z. L. Wang, H. Wu, Bioinspired triboelectric nanogenerators as self-powered electronic skin for robotic tactile sensing, Advanced Functional Materials 30 (2019). doi:10.1002/adfm.201907312.

[11] S. Chen, N. Wu, S. Lin, J. Duan, Z. Xu, Y. Pan, H. Zhang, Z. Xu, L. Huang, B. Hu, J. Zhou, Hierarchical elastomer tuned self-powered pressure sensor for wearable multifunctional cardiovascular electronics, Nano Energy 70 (2020) 104460. doi:10.1016/j.nanoen.2020.104460.

[12] Y. Xiao, Y. Xu, C. Qu, H. Liu, S. Zhang, F. Lin, W. Wu, G. Song, Micro-crack assisted wrinkled pedot: Pss to detect and distinguish tensile strain and pressure based on a triboelectric nanogenerator, Advanced Materials Technologies 7 (2021). doi:10.1002/admt.202100423.

[13] C. Garcia, I. Trendafilova, R. Guzman de Villoria, J. Sanchez del Rio, Self-powered pressure sensor based on the triboelectric effect and its analysis using dynamic mechanical analysis, Nano Energy 50 (2018) 401–409. doi:10.1016/j.nanoen.2018.05.046.

[14] L. Zhao, Q. Zheng, H. Ouyang, H. Li, L. Yan, B. Shi, Z. Li, A size-unlimited surface microstructure modification method for achieving high performance triboelectric nanogenerator, Nano Energy 28 (2016) 172–178. doi:10.1016/j.nanoen.2016.08.024.
